# Development of Multiwalled Carbon Nanotubes/Halloysite Nanotubes Reinforced Thermal Responsive Shape Memory Polymer Nanocomposites for Enhanced Mechanical and Shape Recovery Characteristics in 4D Printing Applications

**DOI:** 10.3390/polym15061371

**Published:** 2023-03-09

**Authors:** Sivanagaraju Namathoti, Manikanta Ravindra Kumar Vakkalagadda

**Affiliations:** School of Mechanical Engineering (SMEC), VIT-AP University, Amaravati 522237, Andhra Pradesh, India

**Keywords:** 4D printing, shape memory polymer, characterization, shape-memory effect

## Abstract

The mechanical and shape-recovery characteristics of 4D-printed thermally responsive shape-memory polyurethane (SMPU) reinforced with two types of reinforcements, multiwalled carbon nanotubes (MWCNTs) and Halloysite nanotubes (HNTs), are investigated in the present study. Three weight percentages of reinforcements (0, 0.5, and 1) in the SMPU matrix are considered, and the required composite specimens are obtained with 3D printing. Further, for the first time, the present study investigates the flexural test for multiple cycles to understand the 4D-printed specimens’ flexural behavior variation after shape recovery. The 1 wt% HNTS-reinforced specimen yielded higher tensile, flexural, and impact strengths. On the other hand, 1 wt% MWCNT-reinforced specimens exhibited quick shape recovery. Overall, enhanced mechanical properties were observed with HNT reinforcements, and a faster shape recovery was observed with MWCNT reinforcements. Further, the results are promising for the use of 4D-printed shape-memory polymer nanocomposites for repeated cycles even after a large bending deformation.

## 1. Introduction

Additive manufacturing (AM), often known as three-dimensional (3D) printing, is a method that generates physical objects layer by layer directly from the digital model data created using computer-aided design (CAD) software. It is growing and becoming more prevalent in the context of 4D printing. The additional dimension relates to “time”, an additional component of traditional 3D printing processes. In 4D printing, a dynamic structure is modeled using 3D printing that can change its physical shape whenever a predefined stimulus is present. In simple terms, the 3D printing of shape-memory materials is called 4D printing. Shape-memory polymers (SMPs) have many advantages, such as low cost, ease of processing, and high shape recovery among all shape-memory materials. Fused deposition modeling (FDM) is a commonly used additive manufacturing technique due to its consistency, reliability, stability, predictability, and low cost. The combination of 3D printing SMPs with the FDM technique presents an ideal opportunity to develop complex 4D-printed structures of varying compositions and properties for specific applications.

Researchers have recently carried out studies on 4D printing various materials and for several applications. The self-healing properties of polyurethane (TPU) and polycaprolactone (PCL) composites were studied in [1]. The final composite exhibited excellent shape recovery and self-healing properties. The effect of 3D printing parameters (height of the layer, speed of the printing, and built plate temperature) on the dimensional stability of 3D-printed specimens using FDM was studied in [2]. Earlier studies from [3] addressed the effect of 3D printing parameters (speed of printing, layer thickness, bed temperature, and nozzle temperature) on the deformation of circular disks using shape-memory polymer. The assessment of PET-G 4D printing as an SMP, including different printing parameters, was studied in [4]. The study showed exemplary shape-memory behavior of PET-G 4D-printed parts regardless of printing condition. PLA/PCL biopolymer with high toughness was prepared in [5] for 4D printing model materials. The results proved that the microphase separation in PLA/PCL-based biopolyurethane could be adjusted by changing the cross-linking ratios.

Thermoplastic shape-memory PEEK reinforced with Cr_20_Ni_80_ (a common electrothermal metallic fiber) was developed for rapid shape recovery in [6], and the heating rate of advanced composites was observed to be 70 times faster than regular water/oil bath. Further, a well-controlled and selective deformation was also achieved from the same. The fabrication of a hybrid soft millirobot using 4D printing technology and a novel hydrogel that can be actuated both thermally and magnetically was developed in [7]. The fabricated millirobot exhibited all deformations successfully in a thermal field. In another study, polybutylene succinate and polylactic acid composite filaments were successfully 4D-printed [8] for photothermal applications. The graphene oxide functionalized filaments of polybutylene succinate and polylactic acid combination exhibited better photothermal properties under near-infrared irradiation. Electroactive polymer composites with PLA/CNT were fabricated [9], and the behavior of complex structures was tested under an electric field. The optimum printing parameters for better shape-memory performance and structural design were studied. The authors of [10] developed thermally responsive and magneto-responsive smart structures fabricated using 4D printing with polylactic acid, thermoplastic polyurethane, and Fe_3_O_4_ particles. The complex structures exhibited shape recovery under a magnetic field, indicating a non-contact shape recovery feature under a magnetic field.

Triple-shape-memory polymer composites using the direct-ink-deposition method were developed in [11]. Further, Fe_3_O_4_ was also added for transformation in a magnetic field. High-strength and high-transition shape-memory polymers were fabricated using the digital light process, direct ink writing, and 3D printing [12]. The 3D-printed technique gave better mechanical properties than other techniques. The strength and toughness of Fe_3_O_4_ and cellulose nanofiber-reinforced magneto-responsive SMP blends were studied in [13]. The 4D printing of complex geometry components (to understand the effect of printing parameters on shape-memory properties) with fused deposition modeling using PLA was studied in [14]. The 4D-printed specimens extruded from shape-memory polymer pellets and mechanical properties were studied in [15]. The authors of [16] proposed a two-pattern design network and studied the performance of the designed pattern in shape fixity. The effect of various printing patterns on the thermomechanical properties of 3D-printed shape-memory polymers was studied in [17]. The authors of [18] proposed 4D auxetic structure systems capable of giving hierarchical motion.

The mechanical properties of shape-memory polymer reinforced with multiwalled carbon nanotubes were studied in [19], and the results showed that mechanical properties and shape recovery improved upon adding reinforcements. A 4D-printed actuator using shape-memory alloy was developed in [20], and the developed shape-memory composite actuator responded well to joule heating. Notably, 1D to 2D and 2D to 3D transformable structures using shape-memory polymers were developed in [21] and successfully demonstrated the developed structures. The authors of [22] successfully obtained 4D-printed specimens using thermally responsive shape-memory polymer and performed shape recovery and voltage experiments. The authors of [23] successfully developed photo-responsive shape-memory devices using shape-memory polymer and carbon black. The authors of [24] studied the mechanical and shape-recovery characteristics of thermally responsive SMPU fabricated via injection molding. The authors of [25] briefly discussed the application of various SMPs and triggering methods. A two-layer structure strategy with ABS shape-memory TPU and PCL-TPU was developed in [26]. The results showed 90% shape fixity for ABS-TPU and 77.42% shape fixity for PCL-TPU. The encapsulated polycaprolactone and thermoplastic polyurethane developed in [27] through 4D printing exhibited a very good stress recovery for the considered combination of PLA and PU. The effect of programming temperature on the PETG specimens fabricated using 4D printing was studied in [28]. Further, the impact of programming conditions on stress recovery was also studied.

Some studies on the 4D printing of thermoplastic shape-memory polymers exist in the literature. Further, only a few studies considered the improvement in the mechanical and shape-recovery characteristics of 4D-printed SMPs. As there is a vast scope for these SMPs to improve mechanical and shape recovery by using reinforcements, the present study aims to fulfill the existing gaps in the field by using two types of reinforcements: MWCNTs and HNTs. (i) MWCNTs, which exhibit exceptional tensile strength, and excellent stiffness, can yield greater toughness for the material and are capable of increasing thermal conductivity. Further, they have a few limitations such as high cost and difficulty in processing; (ii) HNTs have high mechanical strength, thermal stability, and biocompatibility. Various weight percentages of these two reinforcements were considered in a thermally responsive shape-memory polymer matrix using 4D printing (combined with fused deposition modeling using 3D printing technique) and a detailed analysis of mechanical (tensile, flexural, flexural test for repeated cycles, and impact tests) and shape-recovery characteristics (shape-recovery time and % of shape-recovery curves) were studied.

## 2. Materials and Characterization

### 2.1. Details of Shape-Memory Polymer and Specifications of Reinforcements

A thermally responsive shape-memory polyurethane (SMPU) with a glass transition temperature of 56.5 °C and a melting temperature of 163 °C was considered for the present study. In addition, two types of reinforcements, multiwalled carbon nanotubes (MWCNTs) and halloysite nanotubes (HNTs), were considered as reinforcements to obtain nanocomposites with SMPU as the matrix material. The complete specifications and thermophysical properties of the reinforcements used in this study are presented in Table 1.

### 2.2. Processing of Shape-Memory Polymer Nanocomposites and 4D Printing

**Figure 1:**
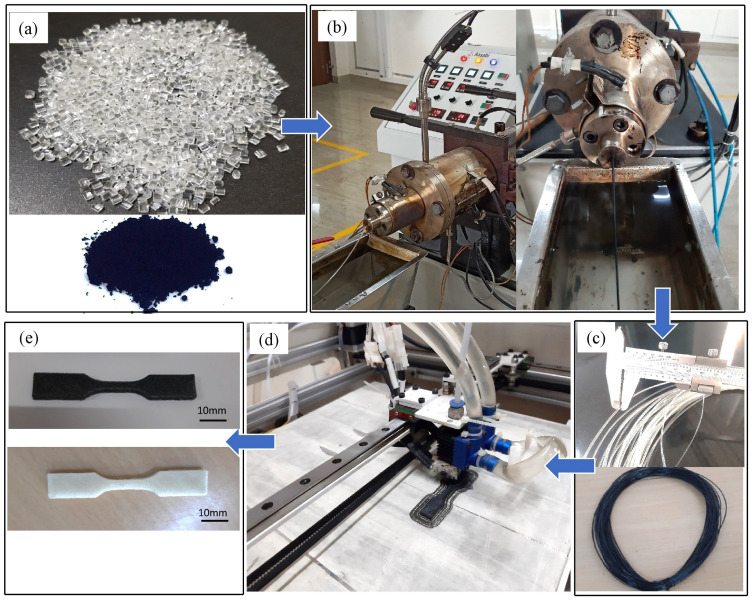
The detailed procedure followed in the present study for 4D printing of pure SMPU and SMPU with reinforcements: (**a**) SMPU pellets and MWCNT reinforcements; (**b**) extraction of composite filaments of required composition; (**c**) extruded filaments of pure SMPU and reinforced with MWCNTs; (**d**) 3D printing of specimen; (**e**) tensile test specimens made of pure SMPU and SMPU reinforced with MWCNTs.

Shape-memory polymer in the form of pellets and MWCNT and HNT reinforcements in powder form were considered for processing and obtaining composite filaments for 3D printing. The complete process involved two major steps: (i) obtaining SMPU matrix filaments with various weight percentages of reinforcements and (ii) the 3D printing of obtained filaments. First, SMPU pellets and reinforcements were dehumidified in a dryer at 80 °C for four hours before extrusion. After dehumidification, the mixtures of SMPU pellets and reinforcements of the required weight percentage were fed into the hopper of a twin screw extruder. Figure 1 shows the complete process of filament extrusion and 3D printing. A screw speed of 40 rpm and melting temperature of 210 °C were used for the filament extrusion in twin screws.

It is critical to choose the best technique for preparing polymer nanocomposites (PNCs) in order to achieve the desired properties for a particular composite material under specified circumstances. PNCs can be made using a variety of techniques, including melt mixing, solution mixing, in situ polymerization, and others. The technique chosen was determined by several variables, including the polymer matrix, the type of filler material, the desired characteristics, and the manufacturing circumstances. Melt mixing involves mixing the polymer and filler in a molten state. Melt mixing is often used for thermoplastic polymers, whereas melt mixing is suitable for low-to-moderate filler loadings, typically up to 10–15% by weight. Thus, melt mixing was adopted in the current work.

Filaments of 1.75 mm diameter with a required weight percentage of reinforcements were used in the SMPU matrix extruded through the output die of the twin screw extruder. The extrude hot filaments were cooled in water immediately. A dual nozzle, fused deposition modeling (FDM)-based 3D printer capable of printing composite filaments was used to print the specimens for tensile, flexural, and impact tests using 3D printing. The composite filaments obtained using a twin screw extruder were dehumidified once again for one hour at 80 °C in a vacuum oven before being fed into input slots of one of the nozzles in a 3D printer. Various parameters were set while performing 3D printing and are shown in Table 2.

### 2.3. Differential Scanning Calorimetry (DSC) Test

DSC analyses were performed on the extruded filaments of various weight percentages of reinforcements obtained from a twin screw extruder after the melt mixing method. DSC analyses were carried out on all the specimens using a simultaneous thermal analyzer (SAT 8000 from PerkinElmer) by considering a sample weight of 25 mg and with a heating rate of 20 °C/min. Tg and Tm were determined by identifying the inflection point (steepest point) with the steps measured in the corresponding zones of the heat flow curve using slope analysis.

### 2.4. Scanning Electron Microscopy (SEM) Test

To understand the distribution and dispersion of reinforcements in the polymer matrix, SEM analyses were performed using scanning electron microscopy (ZEISS EVO10). The surfaces of the reinforced samples were sputter-coated with a thin layer of gold.

### 2.5. Tensile Test

The ASTM D638 (Type-V) tensile specimens were printed using filaments (obtained using a twin screw extruder) of all different weight percentages of MWCNTs and HNTs. Each tensile test was repeated three times. All tensile specimens were tested on a 10 kN universal testing machine at room temperature with a 2 mm/minute cross-head displacement.

### 2.6. Flexural Test

Flexural specimens were obtained from 3D printing for all the weight percentages of SMPU reinforced with MWCNTs and HNTs. Flexural tests were performed on a 10 kN capacity tensile testing machine in a three-point bending mode. The ASTM D790 specimen with dimensions of 125 × 15 × 5 mm^3^ and a gauge length of 80 mm was used to perform flexural tests at room temperature. All flexural tests were performed with a die displacement of 1 mm/minute.

### 2.7. Flexural Tests for Multiple Cycles

**Figure 2:**
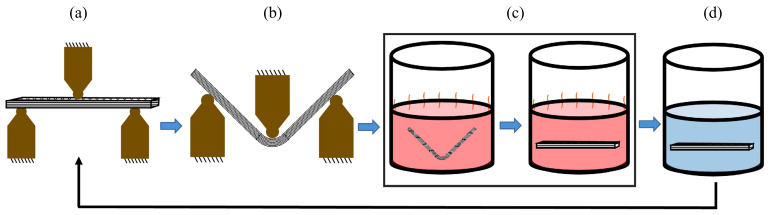
The sequence of the steps involved in a flexural test performed for multiple cycles: (**a**) Un-deformed or initial position of the 4D-printed flexural specimen; (**b**) deformed flexural specimen; (**c**) heating the sample above Tg to recover the permanent shape; (**d**) cooling the sample in cold water to fix the permanent shape and perform the flexural test once again.

The 4D-printed specimens of pure SMPU and reinforced (with MWCNT and HNT) specimens were analyzed using a flexural test for three cycles. Once completing the flexural test (three-point bending test), each specimen’s original shape was recovered in hot water above 80 °C. After the complete recovery of the original shape, the specimen was cooled to room temperature (to fix the permanent shape in cold water), and a flexural test was conducted again. The process of performing the flexural test and recovering the original shape is called a cycle. Figure 2 shows the complete process of a cycle considered in this study.

### 2.8. Shape-Recovery Test

**Figure 3:**
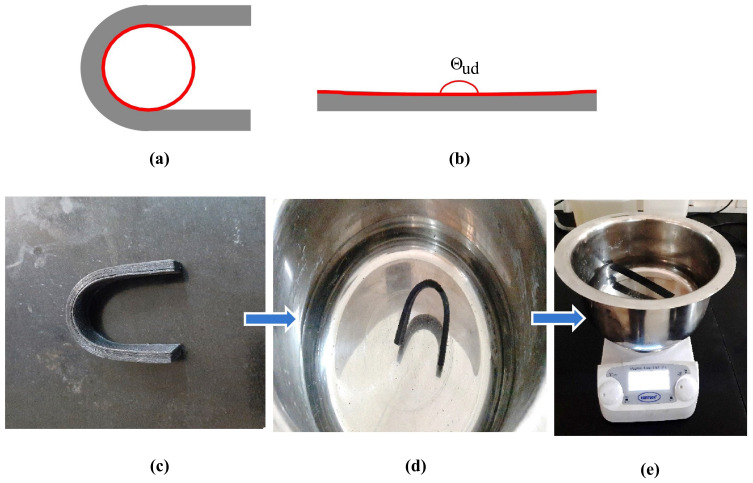
(**a**) Deformed and (**b**) permanent modes of specimens considered for evaluating shape recovery; (**c**) temporary deformed shape; (**d**) kept in a water bath above Tg; (**e**) shape recovery in a water bath.

The specimens of reinforced SMPU of 125 × 15 × 5 mm^3^ were printed for shape-recovery test. The specimens were deformed to a U shape (as shown in Figure 3) after heating to above Tg (in hot water). Once the deformed U shape was achieved, the specimens were kept in cold water (at room temperature of 25 °C) to fix the deformed shape. The specimens in the deformed U shape were again kept in hot water (above Tg), and the time taken for full recovery was noted. The percentage of shape recovery was evaluated using the following equation:

(1) $$\left(1-\frac{\Theta_{ud}-\Theta_{d}}{\Theta_{ud}}\right)\times 100\%$$

where $\Theta_{d}$ is the angle subtended in the deformed mode, and $\Theta_{ud}$ is the angle made in the un-deformed mode. The percentage of shape recovery was calculated at various time frames.

### 2.9. Impact Test

Impact tests were performed using an impact tester to analyze the impact strengths of the specimens made from pure and reinforced SMPU. The specimen for the Charpy impact test was fabricated based on ASTM D6110 with a V-notch of 2.5 mm deep and 45° angle 4D printing. All mechanical tests (tensile, flexural, and impact) were repeated five times as per ASTM standards, and the average values of the results (tensile strength, flexural strength, and impact strength) along with standard deviations are shown in the Results and Discussion section.

## 3. Results and Discussion

### 3.1. Differential Scanning Calorimetry Results

The DSC curves of these samples are presented in [24]. The values of glass transition (Tg) and melting temperatures (Tm) are shown in Table 3. From the results, it can be clearly inferred that the Tg and Tm increased with an increase in the percentage of reinforcements. The main reasons for this include the good dispersion and distribution of the reinforcements and the rigid structure formed through molecular interactions [29]. Further, Tg and Tm values were observed to be slightly high for specimens of MWCNTs.

### 3.2. Scanning Electron Microscopy Results

**Figure 4:**
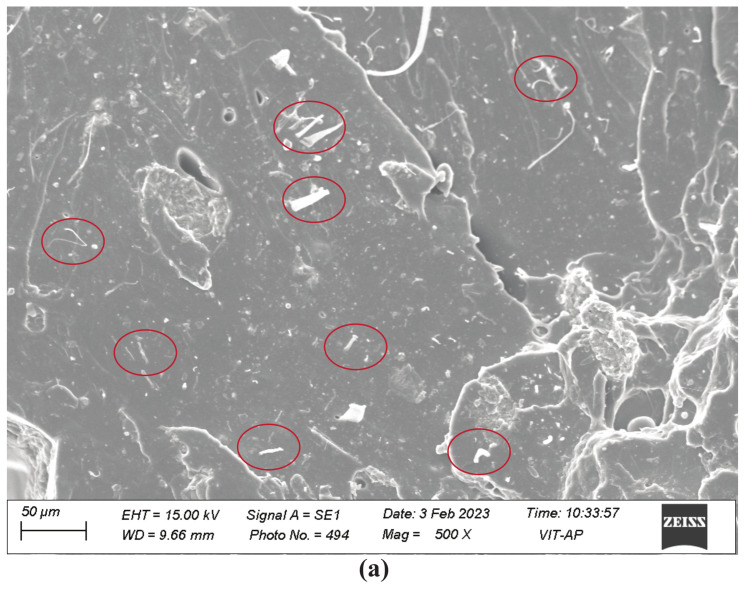

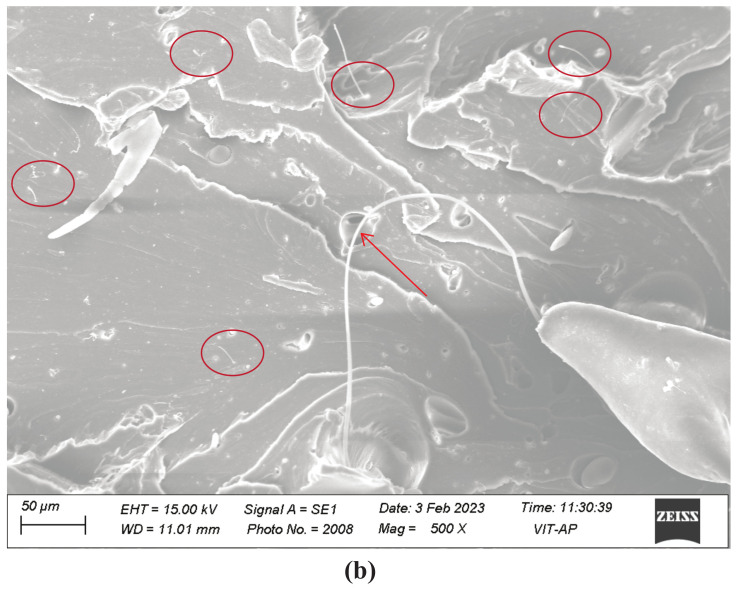
SEM images of 1 wt% (**a**) MWCNT-reinforced and (**b**) HNT-reinforced shape-memory polymer nanocomposites. The red color circled ones indicates dispersed reinforcements.

Figure 4 shows the SEM micrographs of 1 wt% MWCNT and HNT-reinforced SMPU nanocomposites, respectively. It can be inferred that most of the individual MWCNTs and HNTs were fully dispersed in the matrix material. Further, they were randomly aligned and uniformly distributed without any agglomeration. This is evidence for the appropriate dispersion of reinforcements in the matrix material, which could result in improvement in mechanical properties. The observed results are in line with the earlier results reported in [29,30,31].

### 3.3. Results of the Uni-Axial Tensile Test

**Figure 5:**
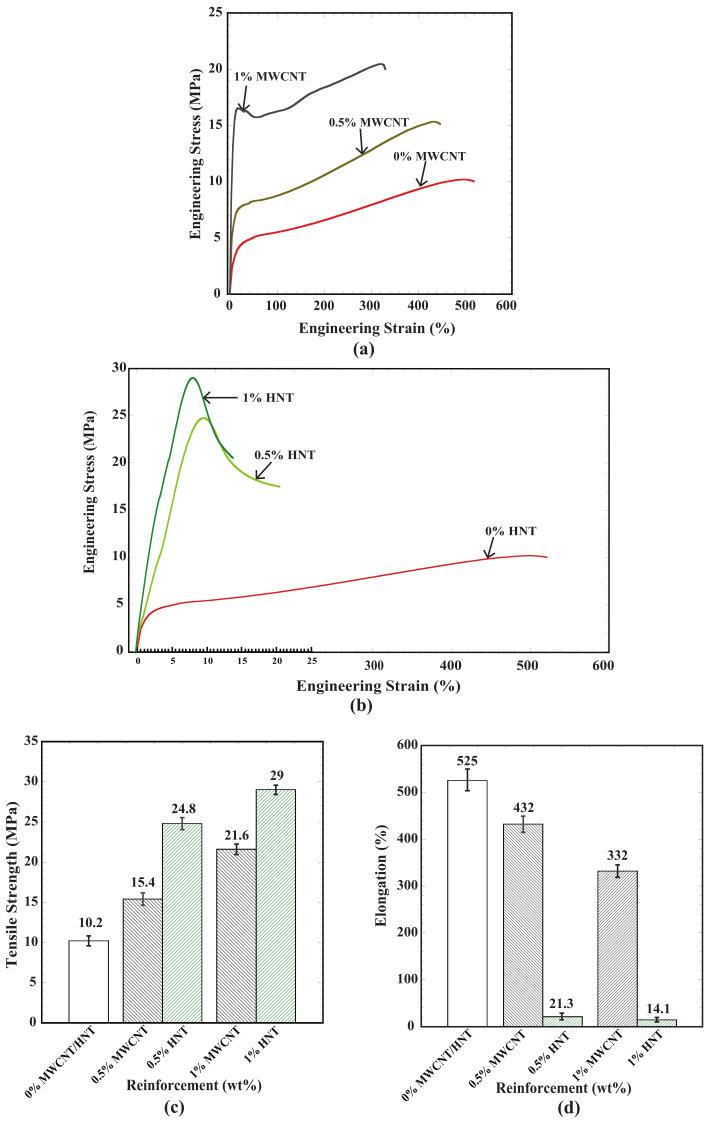
(**a**) Engineering stress vs. strain (%) for SMPU with various wt% of MWCNTs; (**b**) engineering stress vs. strain (%) for SMPU with various wt% of HNTs; (**c**) tensile strength vs. reinforcement % for all specimens; (**d**) Percentage of elongation for all specimens.

Figure 5a,b shows engineering stress vs. the percentage of engineering strain for the SMPU specimens reinforced with MWCNTs and HNTs and fabricated using 4D printing for reinforcement weight percentages of 0, 0.5, and 1. The following findings can be observed from Figure 5a,b: (i) As the % of MWCNTs/HNTs increased, the yield and tensile strengths increased; (ii) for all percentages of reinforcements, strain hardening was observed with an increase in the percentage of strain; (iii) further, a reduction in the percentage of elongation can be observed with an increase in the percentage of reinforcements. Figure 5c,d shows the tensile strength values of all the weight percentages of the reinforcements considered for both types.

The mechanical properties of SMPU specimens reinforced with MWCNTs/HNTs mainly depend on the distribution and orientation of nanoreinforcements in the polymer matrix [29]. A random orientation and uniform and homogeneous dispersion of nanoreinforcements will lead to a greater enhancement of mechanical properties [32]. Further, higher reinforcement–polymer interaction and lower reinforcement–reinforcement interaction will also enhance the mechanical properties. Random orientation and uniform distribution with homogeneous dispersion will lead to better interfacial bonding of reinforcements and matrix materials, leading to better mechanical properties. The same is evident from the SEM images shown in Figure 4. An increase in the restrictions of matrix material polymer chains could be the main reason for the reduction in the percentage of elongation with an increase in reinforcement percentages [33]. This can be more seen in the case of HNTs, resulting in a lower percentage of elongations than MWCNTs. The increase in hard reinforcement content in the polymer matrix led to higher tensile strengths and more rigidity in the polymer matrix chain, which was the main reason for the reduced elongation percentage with the increase in the reinforcement percentage. This seems to be more predominant in the case of HNT reinforcements [33]. All these observations agree with the previous studies of thermoplastic polymer matrix materials reinforced with nanocarbon reinforcements [29,32,33].

At a particular wt% of reinforcement, tensile strength was high for the specimens reinforced with HNTs. At the same time, the elongation percentage was very low for the specimens reinforced with HNTs compared with the specimens reinforced with MWCNTs. At 0.5 wt% of reinforcement, the specimens reinforced with HNTs exhibited 61% higher tensile strength than MWCNTs. By contrast, at 1 wt% reinforcement, HNT-reinforced specimens exhibited 34% higher tensile strength than the specimens reinforced with MWCNTs. On the other hand, there was a considerable difference in the elongation percentage of the specimens reinforced with HNTs and MWCNTs. From Figure 5d, one can observe that the specimens reinforced with HNTs exhibited just 21.3% and 14.1% of elongation at 0.5 and 1 wt% of reinforcements, respectively. In comparison, MWCNT-reinforced specimens exhibited 432 and 332 percentages of elongation. The highest tensile strength of 29 MPa and lowest elongation percentage of 14.1% were observed for 1 wt% HNT-reinforced specimens. The increase in the brittleness of these reinforced composites is due to stronger interfacial interactions between the reinforcements and the SMPU polymer matrix. A similar type of behavior was observed in MWCNT-reinforced non-shape memory thermoplastic polyurethane [34] and MWCNT-reinforced PLA [35].

### 3.4. Flexural Test Results

**Figure 6:**
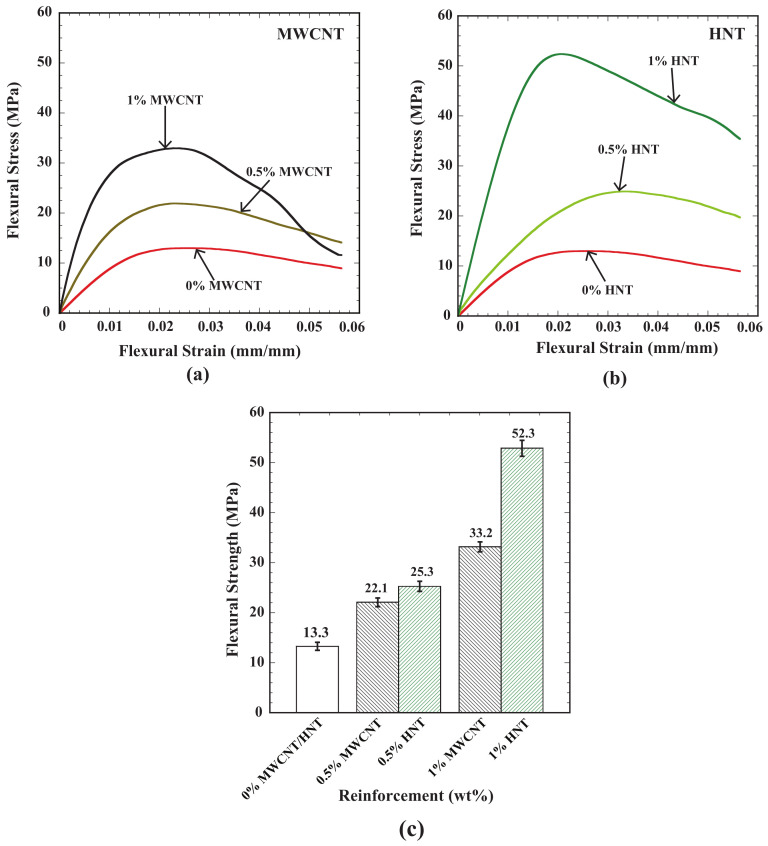
(**a**) Flexural stress vs. flexural strain for SMPU and MWCNT composite 4D-printed specimens; (**b**) flexural stress vs. flexural strain for SMPU and HNT composite 4D-printed specimens; (**c**) flexural strength for all the considered cases of 4D-printed specimens.

Figure 6 shows the result of flexural tests (three-point bending tests) performed on 4D-printed specimens reinforced with MWCNTs and HNTs. Figure 6a,b shows flexural stress as a function of flexural strain for 4D-printed specimens of SMPU reinforced with MWCNTs (Figure 6a) and HNTs (Figure 6b) for three weight percentages of reinforcements (0, 0.5, and 1). Figure 6c shows the flexural strength for all the considered cases of reinforcements and weight percentages.

The following observations can be made from these results: (i) In both types of reinforcements, flexural strength increased with an increase in the weight percentage of reinforcements; (ii) at 0.5 wt% reinforcements, the flexural strength of the specimens reinforced with HNTs exhibited 20% high flexural strength than the specimens reinforced with MWCNTs; (iii) similarly, at 1 wt% reinforcement, the flexural strength of the specimens reinforced with HNTs exhibited 65% high flexural strength than the specimens reinforced with MWCNTs; (iv) with a 0.5 wt% increase in reinforcements (from 0.5 to 1 wt%), there was a 50% increase in flexural strength with MWCNTs; (v) on the other hand, for HNTs, 125% increase in flexural strength was observed while increasing the reinforcement from 0.5 to 1 wt%; and (vi) the highest flexural strength of 52.3 MPa was observed in HNT-reinforced (by 1 wt%) specimens. The random orientation and uniform distribution of the reinforcements in the SMPU matrix added greater strength and enhanced the binding strength of the matrix material under bending loads, which is responsible for the increase in flexural strength with an increase in reinforcement. Similar behavior was also observed in various previous studies of HNT-reinforced polypropylene [36,37].

### 3.5. Flexural Test Results for Repeated Cycles

**Figure 7:**
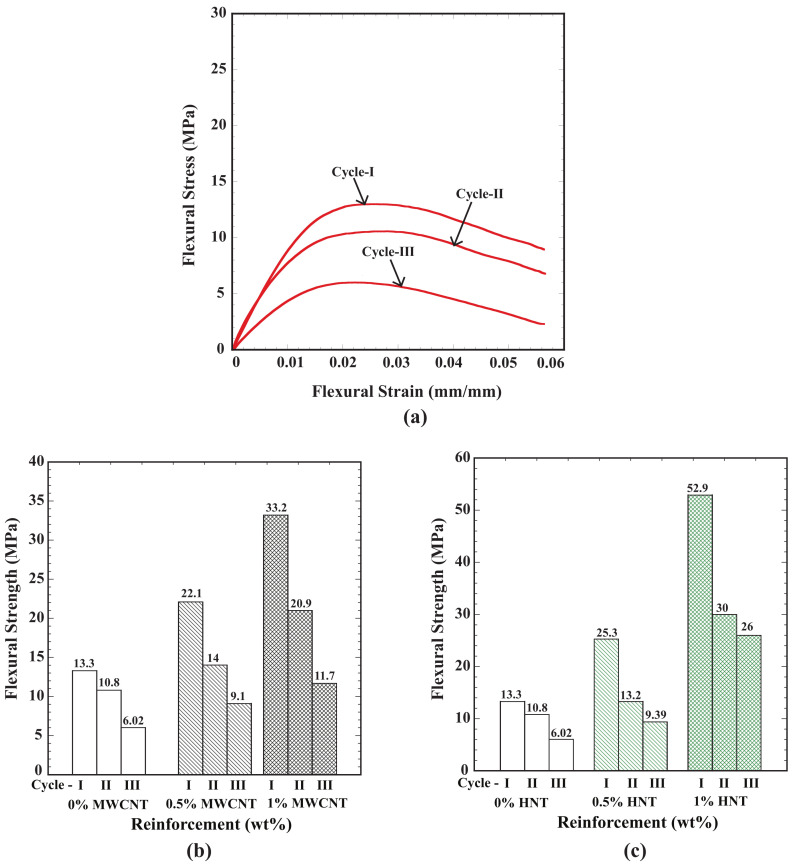
(**a**) Flexural stress vs. flexural strain for pure SMPU 4D-printed specimen for three cycles; (**b**) flexural strength for three cycles of SMPU specimens reinforced in MWCNTs; (**c**) flexural strength for three cycles of SMPU specimens reinforced with HNTs.

After performing the flexural test, all the specimens (for both reinforcements and three weight percentages of reinforcements) were heated above the glass transition temperature to regain their permanent shape. Once they regained their permanent shape, flexural tests were re-conducted. This process was repeated for three cycles. Figure 7a shows the flexural stress as a function of flexural strain for pure SMPU. A gradual reduction in flexural strength was observed as the number of cycles increased, and a reduction of 53% in flexural strength was observed from cycle 1 to cycle 3.

**Figure 8:**
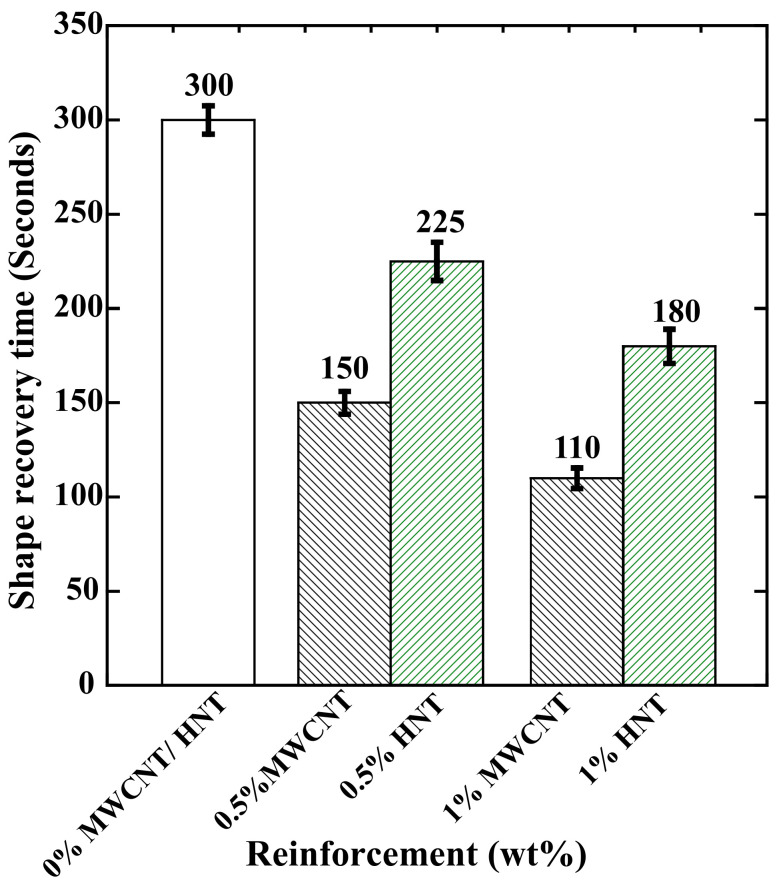
Time required (in seconds) for 100% of shape recovery for 4D-printed pure SMPU and SMPU reinforced with 0.5 and 1 wt% of MWCNTs and HNTs.

The flexural strength of a pure SMPU at cycle 1 in Figure 7b,c shows flexural strength values for the two types of reinforcements (Figure 8b for MWCNTs and Figure 7c for HNTs) for three weight percentages of the reinforcements considered. From Figure 7b, it can be inferred that there was a reduction of 59% in flexural strength from cycle 1 to cycle 3 for the specimens reinforced with 0.5 wt% MWCNTs. By contrast, for the specimens reinforced with 1 wt% MWCNTs, there was a reduction of 64.7% in flexural strength from cycle 1 to cycle 3. It is clear that, as the percentage of MWCNTs increased, there was an increase in the extent of reduction in flexural strength from cycle 1 to cycle 3. In comparison, for the specimens reinforced with HNTs, the corresponding percentage of reduction in flexural strengths for 0.5 and 1 wt% reinforcements from cycle 1 to cycle 3 was found to be 64% and 51%. At 1% of reinforcement, HNT-reinforced specimens exhibited higher flexural strength and a smaller reduction in flexural strength when compared to MWCNT-reinforced specimens.

### 3.6. Shape-Recovery Test Results

Figure 8 shows the time required (in seconds) for 100% shape recovery in the 4D-printed pure SMPU, MWCNT-reinforced, and HNT-reinforced specimens. The following observations can be made from Figure 8: (i) The time required for shape recovery reduced with an increase in the percentage of reinforcements; (ii) at a given weight percentage of reinforcement (for 0.5 or 1), the specimens reinforced with MWCNTs exhibited faster shape recovery than the specimens reinforced with HNTs; (iii) the lowest time taken was 110 s, observed for 1 wt% MWCNT-reinforced specimens.

SMPU involves a 3D molecular network with cross-linking points and connecting segments. The strength of the cross-links ensures permanent shape and shape recovery. The thermoplastic SMPU has polymer chains mainly linked through the formation of a glassy phase. From Figure 8, it is inferred that the shape-recovery time significantly reduced with the increase in reinforcement (for both MWCNTs and HNTs). This is due to improvement in the crystallinity of the soft segments of polymer chains where the reinforcements acted as nucleating agents and increased the cross-linking density with the increase in reinforcement weight percentage, and this was observed with both types of reinforcements. The rise in Tg for the reinforced specimens further increased these factors.

Further, adding reinforcements added a good number of unlocked polymer chains. Additionally, the higher and quick shape recovery in the reinforced specimens is due to the accumulation of higher strain energy, which is possibly due to the uniform distribution of reinforcement, evident from SEM images. The higher stored strain energy helped a more rapid response to release the amount once reaching the glass transition temperature. To conclude, an increase in the crystallinity of the reinforced specimens (which increased unlocked chains) and the accumulation of higher stored strain energy helped the reinforced specimen to recover its shape faster. All these observations are in good agreement with previous studies [9,36,38,39,40].

**Figure 9:**
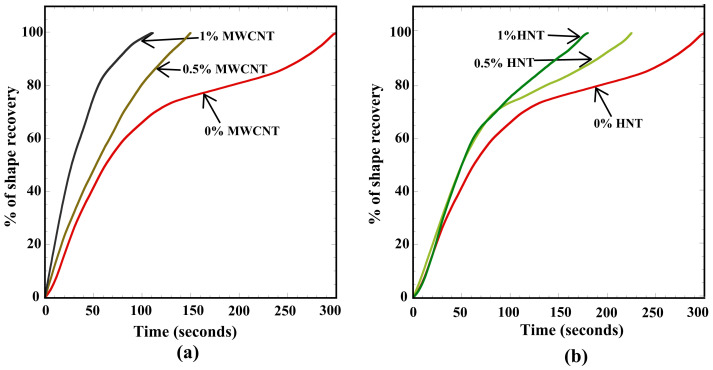
Percentage of shape recovery as a function of time (in seconds) for (**a**) MWCNTs and (**b**) HNTs reinforced 4D-printed SMPU specimens.

Figure 9a,b shows the percentage of shape recovery as a function of time for 4D-printed specimens of SMPU reinforced with 0.5 and 1 wt% of MWCNTs and HNTs. The following important observations can be made from Figure 9: (i) For pure SMPU (0% reinforcement), 70% shape recovery was observed in 120 s, and a sudden reduction in the slope of the shape-recovery curve was observed, indicating a slow shape recovery beyond this point; (ii) higher shape-recovery slopes were observed for 0.5 & 1 wt% SMPU reinforced with MWCNTs. The higher slope for 1 wt% MWCNT-reinforced specimens indicated a faster shape recovery due to higher heat diffusion; (iii) a smaller increase in the slope was observed with an increase in reinforcement weight percentage from 0 to 0.5. At the same time, a larger slope of the shape-recovery curve was observed while increasing the shape-recovery percentage from 0.5 to 1.

**Figure 10:**
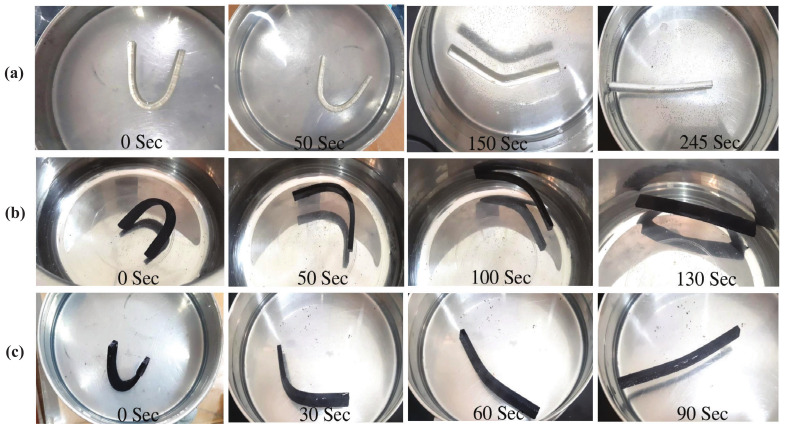
Shape recovery of (**a**) pure SMPU, (**b**) 0.5 wt% MWCNT-reinforced, and (**c**) 1 wt% MWCNT-reinforced specimens at various time frames when placed in the water bath (at 80 °C).

**Figure 11:**
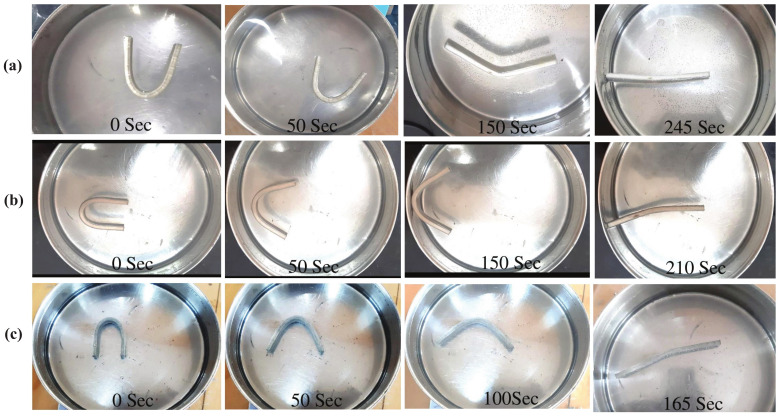
Shape recovery of (**a**) pure SMPU, (**b**) 0.5 wt% HNT-reinforced, and (**c**) 1% HNT-reinforced specimens at various time frames when placed in the water bath (at 80 °C).

Figure 9b shows the shape-recovery curves (as a function of time) for the SMPU specimens reinforced with HNTs. As shown in the shape-recovery curve for 0.5 wt% HNT-reinforced specimens, the shape recovery was faster when compared to pure SMPU specimens. The shape-recovery behavior for 0.5% HNT-reinforced specimens was similar to pure SMPU after 70% of shape recovery. Further, until reaching 70% of shape recovery, the slopes of 0.5 wt% and 1 wt% HNT-reinforced specimens were almost the same, but an improved slope was observed in 1 wt% HNT-reinforced specimens thereafter, which was the reason for faster shape recovery in these specimens. The shape-recovery slopes of HNT-reinforced specimens were lower than those of MWCNT-reinforced specimens due to lower thermal diffusion in HNT-reinforced specimens. Figure 10 and Figure 11 show the shape recovery at various time frames (for 0, 0.5, and 1 wt% MWCNT-reinforced SMPU) during the shape-recovery test. Figure 10a shows the results for pure SMPU, while Figure 11b,c shows the results for 0.5 and 1 wt% MWCNT-reinforced SMPU. Similarly, Figure 11a–c shows the shape recovery at various time frames for 0, 0.5, and 1 wt% HNT-reinforced SMPU. Further, from Figure 9, it can be inferred that the shape recovery was faster in the case of MWCNT-reinforced specimens; this is due to the higher thermal diffusivity of MWCNTs when compared with that of HNTs, as shown in Table 1.

### 3.7. Impact Test Results

**Figure 12:**
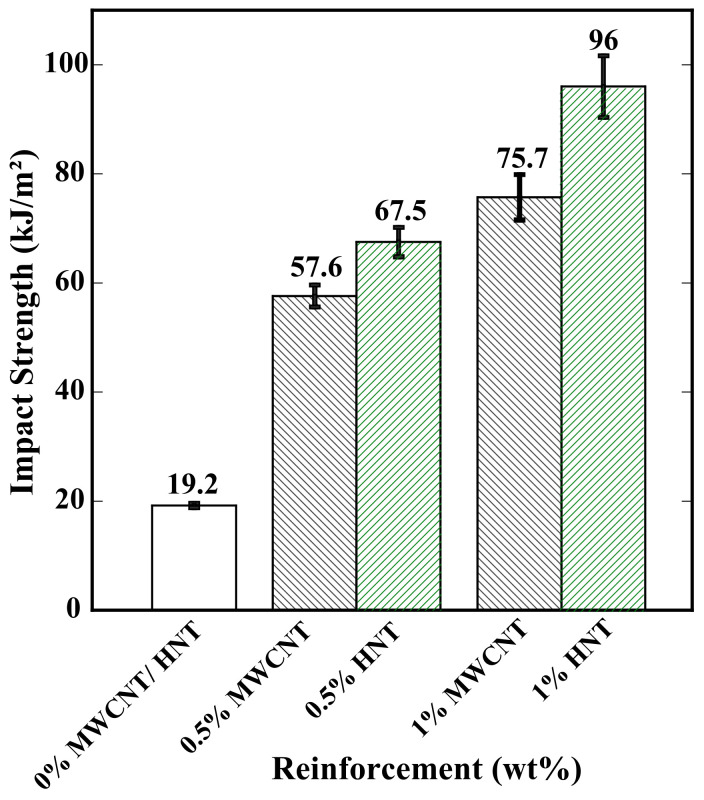
Impact test results of 4D-printed pure SMPU and reinforced specimens.

Figure 12 shows the results of the Charpy tests performed on the 4D-printed specimens of pure SMPU, 0.5, and 1 wt% reinforced with MWCNTs and HNTs. The impact strength is shown on the Y-axis for all the considered cases. The results showed that (i) there was a significant increase in impact strength with an increase in wt% of reinforcement, which means higher energy would be required to break the specimen in an impact test; (ii) for a given weight percentage of reinforcement, HNT-reinforced specimens yielded higher impact strength than the specimens reinforced with HNTs; (iii) with an increase in 0.5 wt% of reinforcement (from 0 to 0.5 wt%), more than 200% increase in impact energy with both types of reinforcements was observed; (iv) at 0.5 wt% of reinforcement, HNT-reinforced specimens yielded 17% higher impact strength than the specimens reinforced with MWCNTs; (v) by contrast, at 1 wt%, HNT-reinforced specimens exhibited 27% higher impact strength than the specimens reinforced with MWCNTs; and (vi) highest impact strength was observed for 1 wt% HNT-reinforced specimens.

## 4. Conclusions

Shape-memory polymer nanocomposites were fabricated and successfully 4D-printed using MWCNTs and HNTs as reinforcements, to enhance mechanical and shape-recovery characteristics for 4D printing applications. Further, we investigated the flexural performance of 4D-printed composites for multiple cycles for the first time. The major conclusions of the experimental results are given below.

- The addition of reinforcements in the SMP matrix significantly affected the tensile strength and percentage of elongation. The HNT-reinforced specimens exhibited higher tensile than the MWCNT-reinforced specimens. At 1 wt%, the HNT-reinforced specimens showed 34% higher tensile strength than the MWCNT-reinforced specimens. At the same time, HNT-reinforced samples exhibited higher brittleness than that MWCNT-reinforced specimens.
- At all the weight percentages considered, HNT-reinforced specimens exhibited greater flexural strength when compared with MWCNT-reinforced specimens. The highest observed flexural strength was at 1 wt% of HNTs, which was about 57% higher than that of 1 wt% MWCNT-reinforced specimens.
- Flexural tests were repeated for three cycles for all the composites. A reduction in flexural strength can be observed after each cycle. Further, the % of this reduction was more in the case of MWCNT-reinforced specimens than that of HNT-reinforced specimens. This indicates better reusability with HNT-reinforced specimens even after a large bending deformation.
- Both reinforcements significantly reduced the shape-recovery time. For all the considered cases, 1 wt% MWCNT-reinforced specimens exhibited a quicker shape-recovery response, which was 38% faster than 1 wt% HNT-reinforced specimens.

As HNT-reinforced specimens exhibited better mechanical properties, and MWCNT-reinforced specimens exhibited better shape-recovery characteristics, the present research facilitates the study of hybrid reinforcements in which one can achieve balance in both mechanical and shape-recovery characteristics.

## Figures and Tables

**Table 1:** Specifications and thermophysical properties of two types of reinforcements considered in the present study.

Reinforcement	MWCNTs	HNTs
Purity (%)	99	99.9
Diameter (nm)	5–20	15
Length (µm)	10	1–15
Thermal conductivity (W/mK)	0.35	0.092
Density (Kg/m^3^)	2100	2540
Specific heat capacity (J/kgK)	550	920
Thermal Diffusivity (mm^2^/s)	0.303	0.039

**Table 2:** Values of variable parameters used to perform 3D printing of various specimens.

Printing Parameter	Value
Layer height (mm)	0.2
Print speed	30 mm/s
Nozzle temperature	220 °C
Print-bed temperature	60 °C
Printing Pattern	45° and 135°

**Table 3:** Tg and Tm values of all the composites considered in the present study [24].

Type of Reinforcement	% of Reinforcement	Tg (°C)	Tm (°C)
Pure SMPU	0	56.5	163
MWCNTs	0.5	63	171
	1	69	182
HNTs	0.5	62	168
	1	66	177

## Data Availability

Not applicable.

## Abbreviations

The following abbreviations are used in this manuscript:

SMPU	Shape-memory polyurethane
MWCNTs	Multiwalled carbon nanotubes
HNTs	Halloysite nanotubes
SMPs	Shape-memory polymers
SMPPs	Shape-memory polymer pellets
$\Theta_{ud}$	Angle of the sample in undeformed/permanent shape
$\Theta_{d}$	Angle of the sample in deformed/temporary shape
