# Correction: Namathoti, S.; Vakkalagadda, M.R.K. Development of Multiwalled Carbon Nanotubes/Halloysite Nanotubes Reinforced Thermal Responsive Shape Memory Polymer Nanocomposites for Enhanced Mechanical and Shape Recovery Characteristics in 4D Printing Applications. *Polymers* 2023, *15*, 1371

**DOI:** 10.3390/polym16101301

**Published:** 2024-05-07

**Authors:** Sivanagaraju Namathoti, Manikanta Ravindra Kumar Vakkalagadda

**Affiliations:** School of Mechanical Engineering (SMEC), VIT-AP University, Amaravati 522237, Andhra Pradesh, India; sivanagaraju.namathoti@gmail.com

The authors wish to make the following corrections to this paper: [1]. There was an error in Section 2.1, the provider of the material is added now. The corrected paragraph is given below:

“A thermally responsive shape-memory polyurethane (SMPU, Pellet type MM-5520 and obtained from SMP Technologies, Tokyo, Japan), with a glass transition temperature of 56.5 °C and a melting temperature of 163 °C, was considered for the present study. In addition, two types of reinforcements, multiwalled carbon nanotubes (MWCNTs, obtained from Shilpa Enterprises) and halloysite nanotubes (HNTs, obtained from Imerys Tableware), were considered as reinforcements to obtain nanocomposites with SMPU as the matrix material. The complete specifications and thermophysical properties of the reinforcements used in this study are presented in Table 1”.

There was an error in the last paragraph of Section 2.2. The details of the 3D printer are included now, and the corrected paragraph is given below:

“Filaments of 1.75 mm diameter, with a required weight percentage of reinforcements, were used in the SMPU matrix extruded through the output die of the twin screw extruder. The extruded hot filaments were cooled in water immediately. A dual nozzle-fused deposition modeling (FDM)-based 3D printer capable of printing composite filaments was used to print the specimens for the tensile, flexural, and impact tests using 3D printing. Various parameters were set while performing 3D printing and are shown in Table 2. A 3D printer (bed volume: 300 × 300 × 300 mm^3^, dual nozzle-type with a 400–500 °C temperature range, and a heat sink nozzle with a water circulation cooling system) obtained from Global 3D labs (Model: Pramaan Custom composite material 3D printer machine) was used for the 3D printing of all specimens in the present study”.

There was an error in Table 1. The range for the diameters of HNTs is included now, and the revised table is given below:

**Table 1:** Specifications and thermophysical properties of two types of reinforcements considered in the present study.

Reinforcement	MWCNTs	HNTs
Purity (%)	99	99.9
Diameter (nm)	5–20	15–50
Length (µm)	10	1–15
Thermal conductivity (W/mK)	0.35	0.092
Density (Kg/m^3^)	2100	2540
Specific heat capacity (J/kgK)	550	920
Thermal diffusivity (mm^2^/s)	0.303	0.039

There was an error in the SEM result, as SEM cannot reveal the dispersion. The corresponding removed sections are:

2.4. Scanning Electron Microscopy (SEM) Test.

3.2. Scanning Electron Microscopy Results.

The authors state that the scientific conclusions are unaffected. This correction was approved by the Academic Editor. The original publication has also been updated.
